# Supplementary material for: Continuing professional development opportunities for Australian endorsed for scheduled medicines podiatrists—What’s out there and is it accessible, relevant, and meaningful? A cross-sectional survey
Source: PLoS One. 2023 Sep 21;18(9):e0289217. doi: 10.1371/journal.pone.0289217 (PMC10513227; doi:10.1371/journal.pone.0289217)
Supplement: S1 File — (PDF) [file pone.0289217.s001.pdf]

You are invited to take part in this research project, *Continuing Professional Development (CPD) requirements of Endorsed for Scheduled Medicines (ESM) – what's out there and is it accessible, relevant and meaningful?* You have been invited because you currently hold endorsement to prescribe medicines.

The Participant Information Sheet below tells you about the research project. It explains the processes involved with taking part.
Please read this information carefully, it will help you to decide if you want to take part in the research. Participation is voluntary. If you do not wish to take part, you do not have to.

Purpose of the research
This project aims to investigate how endorsed for scheduled medicines (ESM) podiatrists are currently meeting their required CPD hours to maintain endorsement with the Podiatry Board of Australia. And, if the learning opportunities currently available to ESM podiatrists are relevant and meaningful to achieve their desired learning goals.

What is involved?
Participation in the project involves completion of an online survey. The survey is anticipated to take no more than 15 minutes. Data collected will be stored securely for five years in safe, password-protected storage on the University of South Australia computers. After which they will be electronically destroyed. Only the investigators listed on this information sheet will have access to the data.

What are the possible benefits of taking part?
While there are no immediate benefits to you of participating, the research findings could inform the current and future development of the podiatry profession, available educational resources and ESM CPD issues and opportunities.

What are the possible risks and disadvantages of taking part?
It is not anticipated that there are any risks to participation in this study

Do I have to take part in this research project?
Participation in any research project is voluntary. If you do not wish to take part, you do not have to, and you can stop completing the survey at any time. If you complete the survey anonymously, once submitted responses cannot be withdrawn. If you decide to take part and provide your name and email but later change your mind, you are free to withdraw your intention to receive further communication regarding the project at any stage by notifying a member of the research team.

What will happen to information about me?
By providing consent, you agree to the research team collecting and using information about/from you for the research project. Any information obtained in connection with this research project that can identify you will remain confidential. This is an internet-based survey. Every effort will be made to ensure that responses are confidential, however the researcher cannot guarantee the confidentiality or anonymity of material transferred by email or the internet. Information collected or used will be stored confidentially. The confidential information you provide may be used for future research in this area by the chief investigator only. It will only be disclosed with your written consent, except as required by law.

The ethical aspects of this research project (#203501) have been approved by the Human Research

Ethics Committee (HREC) of the University of South Australia as required by the Australian government research requirements, specified in the National Statement on Ethical Conduct in Human Research (2007 - updated 2018).

Please read the Participant Information Sheet and indicate your consent below.

* 1. By providing consent you are telling us that you;
- Understand what you have read in the Participant Information Sheet attached
- Consent to take part in the research project
- Consent to the use of your information as described in the Participant Information Sheet.

Do you consent to participating in this survey?

- ◯ Yes
- ◯ No

Demographic details:

* 2. Please select your Podiatry Board of Australia registration category (select one)

- ◯ General podiatrist with endorsement for scheduled medicines
- ◯ Podiatric Surgeon
- ◯ General podiatrist
- ◯ Non-practising

* 3. In what year did you commence practice as a general podiatrist?

* 4. In what year did you obtain endorsment for scheduled medicines or registration as podiatric surgeon?

* 5. Which jurisdiction is your principal place of practice?

- QLD
- NSW
- ACT
- VIC
- TAS
- SA
- NT
- WA

* 6. Do you primarily practise in a regional, rural or remote setting?

- Yes
- No

* 7. In which health setting do you primarily work?

- Private practice
- Community health
- In-patient hospital
- Out-patient hospital
- Aged care
- Disability services
- Podiatric surgeon
- Other (please specify)

* 8. In which health setting do you primarily prescribe medicines?

- Private practice
- Community health
- In-patient hospital
- Out-patient hospital
- Aged care
- Disability services
- Podiatric surgeon
- Other (please specify)

* 9. In which scope of practice do you primarily prescribe medicines?

- High risk foot
- Paediatrics
- Sports
- General podiatry
- Aged care
- Surgery
- Other (please specify)

* 10. I am?

- Male
- Female
- Inter-sex
- Prefer not to answer

CPD habits and engagement

* 11. In the previous 12 month registration period, how many hours of CPD relating to your endorsement for scheduled medicines did you complete?

- ◯ less than 10 hours
- ◯ 10 hours
- ◯ 11-15 hours
- ◯ more than 15 hours

* 12. In the previous 12 month registration period, have you received mentoring to support your prescribing practices?

Definition of mentor: *"Through their knowledge and experience, your mentor provides support for the development of your skills to prescribe scheduled medicines"*
Podiatry Board of Australia, Guidelines: Endorsement for Scheduled Medicines 2018.

- ◯ Yes
- ◯ No

* 13. Which profession was your mentor from?

- ◯ Endorsed for medicines Podiatrist
- ◯ General Practitioner
- ◯ Podiatric Surgeon
- ◯ Nurse Practitioner
- ◯ Pharmacist
- ◯ Other (please specify)

* 14. At any time, have you mentored to completion, a podiatrist undertaking the pathway to become endorsed for scheduled medicines?

(Definition of mentor: as per Podiatry Board of Australia https://www.podiatryboard.gov.au/registration-endorsement/endorsement-scheduled-medicines.aspx)

- ◯ Yes
- ◯ No

* 15. How many mentoring relationships have you taken part in for:

Podiatrists undertaking the endorsed for scheduled medicines pathway (total)?

Podiatrists undertaking the endorsed for scheduled medicines pathway in the previous 12 month registration period?

Podiatrists who are endorsed, but have requested an ongoing mentor realationship?

* 16. Please select the statement that best describes how you plan and complete your CPD:

- ◯ I plan my learning goals first, then find and undertake activities that will meet my learning goals/objectives
- ◯ I participate in CPD activities that take my interest, and then write learning goals reflectively
- ◯ I do not complete learning goals/plan, I complete activities to meet the required number of hours
- ◯ Other (please specify)

* 17. Reflecting on all the endorsed for scheduled medicines CPD activities you completed in the past 12 months, on average, were they;

	Strongly agree	Agree	Neutral	Disagree	Strongly disagree
Easily Accessible?	☐	☐	☐	☐	☐
Affordable?	☐	☐	☐	☐	☐
Meaningful?	☐	☐	☐	☐	☐
Relevant to your scope of practice?	☐	☐	☐	☐	☐
Improved your knowledge?	☐	☐	☐	☐	☐
Translated to a change in your prescribing practice?	☐	☐	☐	☐	☐

* 18. How do you perfer your CPD to be delivered? Select the top 3.

- ☐ Online
- ☐ Face to face
- ☐ Journal articles
- ☐ Self-directed
- ☐ Case studies
- ☐ Peer support
- ☐ Grand rounds
- ☐ Work shadowing/observation
- ☐ Short Course
- ☐ Postgraduate subjects
- ☐ Other (please specify)

* 19. In your opinion, which sectors should be delivering CPD for endorsed for scheduled medicines podiatrists? (select all that apply)

- [ ] Employer
- [ ] APodA
- [ ] Australian College of Podiatric Surgeons
- [ ] University sector
- [ ] 3rd party provider (e.g. fee for service arrangement such as Ausmed platform)
- [ ] Interprofessional/multi-disciplinary prescriber learning events (e.g. with pharmacists, nurse practitioners, GPs etc)
- [ ] Purpose built, one-stop-shop, stand alone repository for non-medical prescribers (e.g website or App containing a range of CPD activities/topics relevant to non-medical prescribers)
- [ ] Pharmaceutical companies
- [ ] All of the above
- [ ] Other (please specify)

* 20. In your opinion, are there improvements to be made in the content, accessibility, relevance and meaningfulness of CPD available to endorsed for scheduled medicines podiatrists?

- ( ) Yes
- ( ) No

* 21. Please detail what, in your opinion, improvements could be made to the relevant sections below:

Content (e.g. topic areas such as disease impacts, prescribing high risk medications, audit skills, special population prescribing, corticosteroids etc)	
Accessibility	
Relevance	
Meaningfulness	
Other	

For the following questions please reflect on the last endorsed for scheduled medicines CPD activity that you completed.

* 22. What was the activity that you participated in?

Title of activity [ ]

Mode of delivery (eg. face to face, online lecture, work shadowing, journal reading, self-audit) [ ]

* 23. What prompted you to participate in this activity? (select all that apply)

- [ ] Clinical situation/interaction
- [ ] Error in prescribing
- [ ] Interest area
- [ ] Self-reflection
- [ ] Prescription audit
- [ ] Broaden scope of prescribing
- [ ] Review of basic/fundamental knowledge
- [ ] CPD that is accessible
- [ ] CPD that is affordable
- [ ] Other (please specify)

[ ]

* 24. Was the activity;

	Strongly agree	Agree	Neutral	Disagree	Strongly disagree
Easily Accessible?	☐	☐	☐	☐	☐
Affordable?	☐	☐	☐	☐	☐
Meaningful?	☐	☐	☐	☐	☐
Relevant to your scope of practice?	☐	☐	☐	☐	☐
Inter or multi-disciplinary	☐	☐	☐	☐	☐

* 25. Did the activity;

	Strongly agree	Agree	Neutral	Disagree	Strongly disagree
Improve your knowledge?	☐	☐	☐	☐	☐
Improve your skills?	☐	☐	☐	☐	☐
Improve your confidence around prescribing?	☐	☐	☐	☐	☐
Translate to a change in your practice?	☐	☐	☐	☐	☐
Improve your communication skills with clients and/or colleagues?	☐	☐	☐	☐	☐
Enable networking, collaboration and relationship building with other prescribing peers?	☐	☐	☐	☐	☐
Result in an departmental/organisational practice change?	☐	☐	☐	☐	☐
Assist in shaping CPD goal development or career/personal development plans?	☐	☐	☐	☐	☐

* 26. Would you recommend this activity to a colleague?

○ Yes

○ No

27. Is there anything else you would like to comment on in relation to CPD for endorsed podiatrists?

28. Thank you for completing the survey. Additional phases of this study will occur in the future. If you would be interested in hearing more about this study or participating further click on this link (opens in a new browser) to enter your details. https://www.surveymonkey.com/r/MGTJNSZ.

To submit your responses to the survey click DONE before leaving the page.
