# Supplementary material for: Continuing professional development opportunities for Australian endorsed for scheduled medicines podiatrists—What’s out there and is it accessible, relevant, and meaningful? A cross-sectional survey
Source: PLoS One. 2023 Sep 21;18(9):e0289217. doi: 10.1371/journal.pone.0289217 (PMC10513227; doi:10.1371/journal.pone.0289217)
Supplement: S1 Table — (PDF) [file pone.0289217.s002.pdf]

Checklist for Reporting Results of Internet E-Surveys (CHERRIES)

Checklist Item	***Explanation***	***Line number***
Describe survey design	We conducted a descriptive, cross-sectional, anonymous online survey of endorsed for scheduled medicines (ESM) podiatrists. Convenience sampling was utilised.	Line 124<br>Line 135
IRB approval	Ethics approval was obtained from the Human Research Ethics Committee from the University of South Australia (Protocol number 203501). The study was performed in accordance with the ethical standards of the Declaration of Helsinki.	Line 125
Informed consent	A detailed participant information sheet was displayed prior to the survey specifying the purpose of the study, approximate time to complete the survey, arrangements for the protection of personal information collected and data storage, and particulars of the investigative team. Question one of the survey was mandatory and required all participants to provide informed digital consent before proceeding.	Line 143<br>See appendix 1, page 1.
Data protection	This study was an anonymous survey.<br>At the end of the survey, participants had the option to enter a subsequent survey and leave their name and email address if they consented to the research team contacting them in the future about the topic. This data was not connected to responses provided in the original survey, therefore ensuring anonymity was maintained.<br>Data is stored as detailed in the participant information sheet. Data will be kept for 5 years, in password-protected storage on the University of South Australia's computers, at which time they will be electronically destroyed. Only named investigators have access to collected data.	Line 124<br>See appendix 1, page 1.
Development and testing	Data were collected using a purpose-built survey.<br>The survey was pilot tested over two rounds by a convenience sample of five allied health clinicians (two podiatrists, an optometrist, pharmacist, and speech pathologist) who were asked to provide feedback regarding the structure, functionality, ambiguity, and face validity of the questions. The podiatrists involved in pilot testing were excluded from the final survey.	Line 149<br>Line 196
Open survey versus closed survey	The survey was presented as an open survey.	N/A
Contact mode	Potential participants were alerted to the study via several means. The research team utilised their extensive professional networks with invitations to participate and the survey URL link distributed via email.<br>An advertisement was circulated to the Australasian College of Podiatric Surgery (ACPS) email distribution list.	Line 136<br><br>Line 138
Advertising the survey	Potential participants were alerted to the study via several means. The research team utilised their extensive professional networks with invitations to participate and the survey URL link distributed via email and social media	Line 136

	posts (Twitter™ and Facebook™). An advertisement was circulated to the Australasian College of Podiatric Surgery (ACPS) email distribution list. Participants were also encouraged to share the survey URL link amongst their ESM podiatry colleagues.<br><br>Wording of emails sent directly to known contacts and the ACPS were as follows:<br><br>Dear ……,<br><br>Helen Banwell, Kristin Graham, Jacinta Johnson and I are investigating CPD available to endorsed for scheduled medicines Podiatrists, and your input as a prescriber would be appreciated.<br><br>The online survey will take no more than 15 minutes to complete and will help us understand the in's and out's of CPD for endorsed Pods and Podiatric Surgeons.<br><br>This research has been approved by UniSA HREC 203501<br><br>For full details of the study, Participant Information Sheet and to start the survey, click the button below.<br><br>**Survey closes 30 September 2021.** Please feel free to distribute the link amongst your prescribing Podiatry networks.<br><br>Many thanks for your participation<br><br>Saraid Martin<br><br>Saraid Martin | B.Pod| Masters by Research Candidate: Allied Health & Human Performance<br>University of South Australia | City East Campus | Adelaide SA 5000 |saraid.martin@mymail.unisa.edu.au | www.unisa.edu.au | <br>Begin Survey<br><br>Twitter™ post from S.Martin account:<br>Call out to all Aust endorsed Pods & Pod Surgeons. We need your help to understand the in's and out's of CPD to support prescribing. For more info & to complete the survey click below. Your feedback is important! (survey link)	

	Facebook™ post from S.Martin account:<br>Pod peeps – please consider completing this survey to support my Masters and help understand the area more. (survey link) Australian Endorsed for Scheduled Medicines Podiatrists.	
Web/E-mail	A descriptive, cross-sectional, anonymous online survey (Survey Monkey ™, California, USA)	Line 124
Context	Not applicable	N/A
Mandatory/voluntary	All Australian podiatrists with an endorsement for scheduled medicines and podiatric surgeons (N = 167 as of September 2021), were eligible for enrolment in this voluntary study	Line 133
Incentives	No incentives were offered to potential participants	Line 140
Time/Date	The online survey was open for five weeks between August and September 2021	Line 206
Randomization of items or questionnaires	Randomisation was not utilised to ensure the sensical flow of the survey and adaptive questioning could occur.	N/A
Adaptive questioning	Question logic was turned on to reduce the number of items posed to participants.	Line 202
Number of Items	The final questionnaire consisted of 28 items over three sections (Appendix 1).<br>Section 1: Focused on participant characteristics. This page had eight items and was the maximum number of items on one page.	Line 152<br>See appendix 1, page 5
Number of screens (pages)	As question logic was turned on, the number of pages displayed to participants was dictated by their responses. The maximum number of pages was ten, with the minimum number two.	See appendix 1
Completeness check	Key questions required mandatory answers to progress in the survey	Line 201
Review step	Questions were presented sequentially with participants able to navigate back within the browser to amend answers prior to completion.	Line 200
Unique site visitor	Not available	N/A
View rate (Ratio of unique survey visitors/unique site visitors)	Not available	N/A
Participation rate (Ratio of unique visitors who agreed	A total of 42 survey responses were received.	Line 217

to participate/unique first survey page visitors)		
Completion rate (Ratio of users who finished the survey/users who agreed to participate)	Nine responses were excluded as participants were not ESM podiatrists or podiatric surgeons (n = 6) or failed to respond to questions outside of personal demographics (n = 3). The remaining 33 responses included in data analysis, represented 20% (n = 33/167) of the eligible population of ESM podiatrists and podiatric surgeons.	Line 217
Cookies used	To prevent more than one entry from individuals, the multiple responses option within SurveyMonkey ™, which uses cookies, was turned off.	Line 141
IP check	IP address was not collected.	N/A
Log file analysis	No other techniques were used to analyse log files	N/A
Registration	Not applicable. This was an open survey	N/A
Handling of incomplete questionnaires	A total of 42 survey responses were received. Nine responses were excluded as participants were not ESM podiatrists or podiatric surgeons (n = 6) or failed to respond to questions outside of personal demographics (n = 3). The remaining 33 responses included in data analysis, represented 20% (n = 33/167) of the eligible population of ESM podiatrists and podiatric surgeons.<br><br>Question responses were made mandatory to increase completeness rate however, as question logic was turned on, not all questions required every participant to answer.<br><br>Some survey participants skipped mandatory questions resulting in incomplete survey responses being received. Those that had progressed past the demographic questions were included for analysis (n=33). Mandatory questions not involved in question logic that did not receive n=33 responses were: Q16 (n=31); Q17 (n=31); Q22 - 26 (n=28).	Line 217<br><br>Line 201<br><br>Line 221
Questionnaires submitted with an atypical timestamp	No time stamp cut-off period was implemented for participants to complete the survey.	Line 207
Statistical correction	Data were exported from SurveyMonkey™ into a Microsoft Excel™ workbook. Descriptive data (age, years of registration and years of endorsement) were reviewed for normality. Responses to closed-ended questions were summarised using descriptive statistic calculations within Excel™.	Line 210

This checklist has been modified from Eysenbach G. Improving the quality of Web surveys: the Checklist for Reporting Results of Internet E-Surveys (CHERRIES). J Med Internet Res. 2004 Sep 29;6(3):e34 [erratum in J Med Internet Res. 2012; 14(1): e8.]. Article available at https://www.jmir.org/2004/3/e34/; erratum available https://www.jmir.org/2012/1/e8/. Copyright ©Gunther Eysenbach. Originally published in the Journal of Medical Internet Research, 29.9.2004 and 04.01.2012.

This is an open-access article distributed under the terms of the Creative Commons Attribution License (https://creativecommons.org/licenses/by/2.0/), which permits unrestricted use, distribution, and reproduction in any medium, provided the original work, first published in the Journal of Medical Internet Research, is properly cited.
