# Supplementary material for: Continuing professional development opportunities for Australian endorsed for scheduled medicines podiatrists—What’s out there and is it accessible, relevant, and meaningful? A cross-sectional survey
Source: PLoS One. 2023 Sep 21;18(9):e0289217. doi: 10.1371/journal.pone.0289217 (PMC10513227; doi:10.1371/journal.pone.0289217)
Supplement: S2 Table — (PDF) [file pone.0289217.s003.pdf]

Likert scale analysis

Question 17 (n=31)

Reflecting on all the endorsed for scheduled medicines CPD activities you completed in the past 12 months, on average, were they;

Parameter	Median	Quartile 1	Quartile 3	IQR
Easily accessible?	4	3	4	1
Affordable?	4	3	4	1
Meaningful?	4	3	4	1
Relevant to your scope of practice?	4	3	4	1
Improved your knowledge?	4	3	4	1
Translated to a change in your prescribing practice?	4	3	4	1

For the following questions please reflect on the last endorsed for scheduled medicines CPD activity that you completed.

Question 24 (n=28)

Was the activity;

Parameter	Median	Quartile 1	Quartile 3	IQR
Easily accessible?	5	4	5	1
Affordable?	4.5	4	5	1
Meaningful?	4	3.75	5	1.25
Relevant to your scope of practice?	4	4	5	1
Inter or multidisciplinary?	4	3	5	2

Question 25 (n=28)

Did the activity;

Parameter	Median	Quartile 1	Quartile 3	IQR
Improve your knowledge?	4	3	5	2
Improve you skills?	4	3	4	1
Improve your confidence around prescribing?	3	3	4	1
Translate to a change in prescribing practice?	3	3	4	1
Improve your communication skills with clients and/or colleagues?	3	3	4	1
Enable networking, collaboration and relationship building?	3.5	2	4	2
Result in an departmental/organisational practice change?	2	2	3	1
Assist in shaping CPD goal development or career/personal development plans?	3	3	4	1
